# Functional meta-omics provide critical insights into long- and short-read assemblies

**DOI:** 10.1093/bib/bbab330

**Published:** 2021-08-27

**Authors:** Valentina Galata, Susheel Bhanu Busi, Benoît Josef Kunath, Laura de Nies, Magdalena Calusinska, Rashi Halder, Patrick May, Paul Wilmes, Cédric Christian Laczny

**Affiliations:** Luxembourg Centre for Systems Biomedicine, 7, avenue des Hauts-Fourneaux, Esch-sur-Alzette L-4362, Luxembourg; Luxembourg Centre for Systems Biomedicine, 7, avenue des Hauts-Fourneaux, Esch-sur-Alzette L-4362, Luxembourg; Luxembourg Centre for Systems Biomedicine, 7, avenue des Hauts-Fourneaux, Esch-sur-Alzette L-4362, Luxembourg; Luxembourg Centre for Systems Biomedicine, 7, avenue des Hauts-Fourneaux, Esch-sur-Alzette L-4362, Luxembourg; BioSystems and Bioprocessing Engineering, Luxembourg Institute of Science and Technology, Rue du Brill 41, Belvaux L-4422, Luxembourg; Luxembourg Centre for Systems Biomedicine, 7, avenue des Hauts-Fourneaux, Esch-sur-Alzette L-4362, Luxembourg; Luxembourg Centre for Systems Biomedicine, 7, avenue des Hauts-Fourneaux, Esch-sur-Alzette L-4362, Luxembourg; Luxembourg Centre for Systems Biomedicine, 7, avenue des Hauts-Fourneaux, Esch-sur-Alzette L-4362, Luxembourg; Luxembourg Centre for Systems Biomedicine, 7, avenue des Hauts-Fourneaux, Esch-sur-Alzette L-4362, Luxembourg

**Keywords:** third-generation sequencing, long reads, Oxford Nanopore Technologies, hybrid assembly, functional omics, meta-omics

## Abstract

Real-world evaluations of metagenomic reconstructions are challenged by distinguishing reconstruction artifacts from genes and proteins present *in situ*. Here, we evaluate short-read-only, long-read-only and hybrid assembly approaches on four different metagenomic samples of varying complexity. We demonstrate how different assembly approaches affect gene and protein inference, which is particularly relevant for downstream functional analyses. For a human gut microbiome sample, we use complementary metatranscriptomic and metaproteomic data to assess the metagenomic data-based protein predictions. Our findings pave the way for critical assessments of metagenomic reconstructions. We propose a reference-independent solution, which exploits the synergistic effects of multi-omic data integration for the *in situ* study of microbiomes using long-read sequencing data.

## Background

Third-generation, single-molecule, long-read (LR) sequencing is considered to be the next frontier of genomics [1], especially in the context of studying microbial populations [2, 3]. Given the ability to attain read lengths in excess of 10 Kbp [4] and continuous sequence accuracy improvements [5], LR sequencing has been recommended for its ability to resolve GC-rich regions, complex and repetitive loci, and segmental duplications in genomes, among others [4]. However, LR applications to study microbiomes have focused on genome assemblies [6, 7], closing a select few bacterial genomes [8], haplotype and strain resolution [9] as well as mock (low diversity) communities [3]. Stewart *et al*. [10] recently were among the first to demonstrate the utility of using LRs for improving upon existing protein databases owing to a large collection of novel proteins and enzymes identified, thereby hinting at the benefits of LRs also for functional microbiome studies.

Single base accuracy of raw LRs remains lower—for now—compared with short-read (SR) methodologies [11]; however, Nanopore LR quality is steadily increasing. Several approaches including assembly based and/or including polishing steps have been developed [11–13] to increase the reconstruction accuracy. The impact of remnant errors in LR assemblies on gene calling and thereby protein prediction was recently highlighted by Watson *et al*. [14]. Hybrid (HY) assembly methods [15, 16] using both SRs and LRs have been proposed to further reduce the error rates compared with LR-only assemblies. Although Watson *et al*. [14] showed that insertions/deletions (indels) play a critical role in microbial protein identification, the overall impact of assembly methods on understanding the functional potential of microbial communities is lacking.

Here, we demonstrate that metagenomic assembly approaches (SR, LR and HY) not only differ markedly in their overall assembly performance, but also in the inferred functional potential. We reveal the effects of the assembly approach on predicted genes and proteins in samples ranging from low to high diversity, from mock communities to human fecal and rumen metagenomes. We find proteins which are exclusive to respective assemblers and demonstrate using metatranscriptomic and metaproteomic data available for the human fecal sample the synergistic effect on protein verification. Our results indicate that irrespective of sample diversity, the sequencing and assembly strategies impact downstream analyses and that complementary omics are a key for functional analyses of microbiomes.

## Results and discussion

To understand how sample diversity, assembly quality and assembly approach are linked, we assembled published metagenomic (metaG) data from a mock community (Zymo), a natural whey starter culture (NWC), a cow rumen sample (Rumen) and a novel metagenomic dataset from a human fecal sample (GDB). The latter was complemented with metatranscriptomic (metaT) and metaproteomic (metaP) data. The samples’ diversity ranged from low (Zymo and NWC) to high (GDB and Rumen). As expected [10], the assembly approach strongly affected the quality of the resulting assembly (Supplementary Figure S1). LR and HY approaches generated fewer contigs with a larger N50 value, supporting the added value of these approaches for achieving increased contiguity and decreased redundancy, thereby also improving the recovery of metagenome-assembled genomes [8]. However, other assembly metrics, e.g. the total assembly length, varied between the samples and assembly types. The metaG read mapping rate (including multi-mapped reads), as a proxy of data usage, was unaffected by the assembler choice when considering all contigs, though the values for the LR assemblies were a bit lower than for SR or HY assemblies of the high-diversity samples (GDB and Rumen). However, the mapping rates dropped markedly in SR assemblies, especially in NWC and Rumen, when filtering out contigs below 5000 bp (Supplementary Figure S2). In GDB, we observed higher metaT read mapping rates in SR and HY assemblies than in LR assemblies. This indicates the complementarity of SR and LR data. The mapping rates decreased considerably in SR assemblies when removing short contigs (Supplementary Figure S3), suggesting the presence of expressed genes located on these contigs. This demonstrates the loss of information when contigs below a certain threshold are removed, which is frequently done in metagenomic studies.

**Figure 1:**
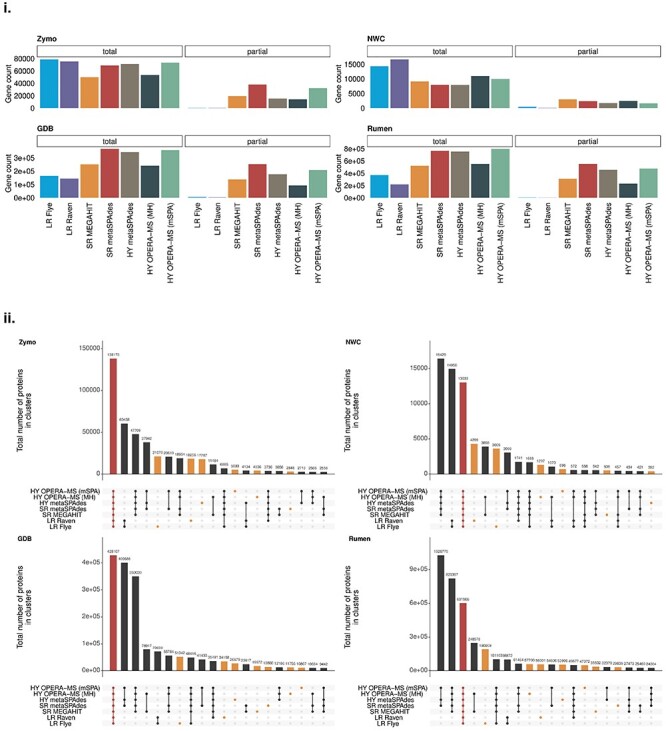
Discrepancy and uniqueness of predicted proteins in assemblies. (i) Number of proteins (total and partial) predicted by Prodigal in each assembly and sample. The color corresponds to the metagenomic assembly approach. (ii) Number of shared predicted proteins which were clustered using MMSesq2 per sample. Each protein cluster was labeled by the combination of assembly tools represented by the clustered proteins (i.e. the assembly where these proteins originated from). The depicted number of shared proteins per assembly tool combination is the total protein count over all associated clusters. Top 20 combinations are shown. The number of proteins found in clusters representing all assembly tools is highlighted in red; the number of proteins exclusive to an assembly is highlighted in orange.

Comparing assemblies pairwise, we observed higher dissimilarities between the LR and SR/HY assemblies than within the latter groups. In addition, OPERA-MS-based HY assemblies clustered together with the SR assemblies on which they were based (Supplementary Figure S4). To assess functional potential overlap between the different assembly approaches, we studied the proteins found in the individual metagenomes. The overall number and quality of predicted proteins was highly influenced by the assembly approach. In highly diverse metagenomes (GDB and Rumen), the total number of proteins in SR and HY assemblies was higher (by a factor of up to 3.67) than in LR assemblies (Figure 1i). However, throughout all samples, the SR and HY approaches produced more partial proteins [incomplete coding sequence (CDS)]. Since SR and HY assemblies may be more fragmented, the polished LR assemblies may have led to an improved recovery of genes. We clustered the predicted protein sequences and found a considerable number of proteins exclusive to individual assemblies. We also found proteins that were shared within a subset of the assemblies only, and that increased sample diversity resulted in an overall increase in the number of exclusive proteins (Figure 1ii).

**Figure 2:**
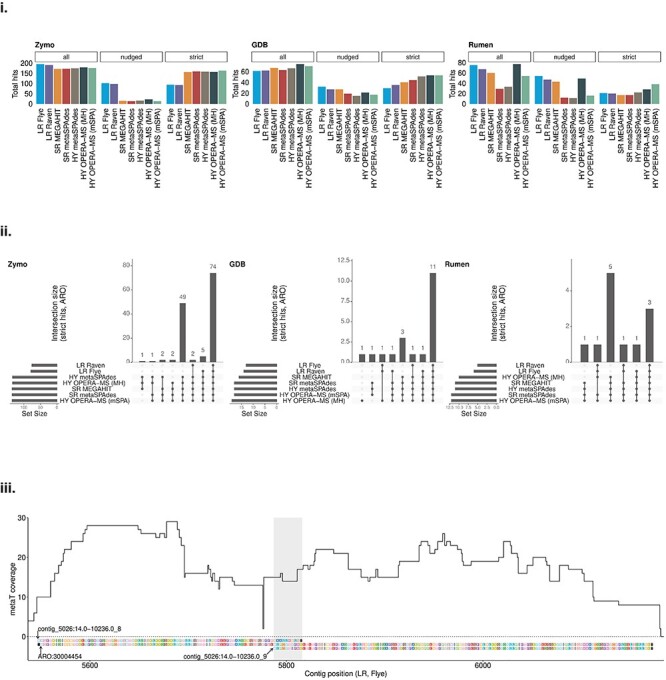
Assembly effects on antimicrobial resistance gene identification. (i) Number of hits (‘all’, ‘strict’ and ‘nudged’) for each assembly and sample when searching the assembly proteins in the CARD database using RGI. The NWC sample is not shown because no hits were found in any of its assemblies. ‘Nudged’ hits are loose hits (distant/incomplete homolog) flagged as such by RGI; the remaining hits are ‘strict’ hits. (ii) Number of Antibiotic Resistance Ontologies (AROs), which were covered by ‘strict’ RGI hits by different assemblies per sample. The bar plot shows the number of shared AROs per assembly tools combination. (iii) Metatranscriptomic (metaT) coverage of the two coding sequences (CDSs) from the long-read (LR) assembly constructed with Flye and having a ‘nudged’ RGI hit to ARO 3004454 (a chloramphenicol acetyltransferase) in the GDB sample. The *x*-axis represents the contig coordinates and the *y*-axis the metaT coverage. The amino acid sequence of the two CDSs and the ARO is included in the plot.

As reported previously by Watson *et al.* [14], errors in LR assemblies can have an impact on the predicted proteins. To evaluate how the sample diversity might affect this, we mapped the predicted proteins against the UniProtKB/TrEMBL nonredundant (nr) protein database and computed the query-to-subject length ratio [10]. In all cases, the density distribution of the ratio values had two peaks (below 0.5 and around 1), though the differences between the assembly methods varied across the samples (Supplementary Figure S5). Considering the above findings and despite multiple rounds of polishing, we cannot disregard the impact of (remnant) errors in LRs affecting the results. Furthermore, the results may also be affected by the sequencing depth and gene prediction methods. One also has to account for the microbial composition per sample, given that a large proportion of proteins from the Rumen sample might not have homologs within the UniProtKB/TrEMBL nr database.

Due to the differences in annotations, which we found to be exclusive to individual assembly approaches, we subsequently studied the effect of assembler choice on two well-defined, functionally relevant classes of genes: ribosomal RNA (rRNA) and antimicrobial resistance (AMR) genes. Overall, the total number of rRNA genes recovered by LR and HY approaches was higher across all samples. Within the archaeal and bacterial domains, LR and HY assemblies led to the prediction of more complete genes compared with SR (Supplementary Figure S6). Our findings are in line with Overholt *et al.* [17] and Xie *et al*. [18], who reported improved recovery and contiguity of rRNA genes, and improved gene completeness, respectively. When analyzing AMR proteins and focusing only on ‘strict’ hits (i.e. excluding loose hits flagged as ‘nudged’ by the Resistance Gene Identifier (RGI) tool, see Methods), HY assemblers were more adept at reconstructing these proteins compared with either SR or LR. Moreover, LR assemblies contained more ‘nudged’ hits than SR or HY assemblies, suggesting that error rates or other factors might have affected the reconstruction of some AMR genes (Figure 2i). Interestingly, we did not identify any AMR hits in the NWC metagenome, possibly due to it being a food-grade additive [19]. When comparing the overlap of the Antibiotic Resistance Ontology (ARO) terms covered by ‘strict’ hits, we found that some AROs were only identified in SR and HY assemblies, but not in LR, whereas no AROs were found in LR assemblies only (Figure 2ii).

To validate the exclusive AROs found in SR and HY assemblies, we assessed metaT and metaP coverage of the corresponding genes and proteins in the GDB sample. The genes mapping to the exclusive AROs had an average metaT coverage above 14× in the SR and HY assemblies, suggesting that these genes are expressed *in situ*; the few ‘nudged’ hits were below 6× (Supplementary Table S1). However, we did not identify these genes in the metaP data potentially due to low expression levels, variation in extraction protocols or posttranslational modifications affecting the peptide/proteomic recovery. Though no ‘strict’ hits were found in LR assemblies, some of their ‘nudged’ hits had an average metaT coverage above 10×. To understand why these seemingly expressed genes obtained only a partial hit, we focused on two ‘nudged’ hits assigned to ARO 3004454 (a chloramphenicol acetyltransferase) in the LR assembly constructed with Flye. We found that the CDSs were located on the same contig and had an overlap of 29 bp. The sequence alignments showed that the respective genes represent two fragments of the true CDS (corresponding to ARO 3004454) most likely created by an indel, which introduced a frameshift and also a premature stop codon. This finding was also supported by the metaT coverage extending beyond the stop codon of the first CDS until the end of the second CDS with a single drop in coverage before the putative indel (Figure 2iii). In both the SR and HY assembly approaches, ARO 3004454 obtained a ‘non-nudged’ hit, indicating a potentially complete gene sequence.

To identify high-confidence proteins without the need for a reference, we first considered proteins and protein clusters found in all assemblies, which represented 22.97% of the proteins and 8.54% of the protein clusters. These included genes reconstructed by the different and independent assembly approaches, thus lending mutual support. We then used the complementary metaT data and included all additional proteins with an average metaT coverage ≥10× and the corresponding protein clusters. This doubled the number of high-confidence protein clusters (17.63%) and increased the percentage of high-confidence proteins to 30.32%.

## Conclusions

We show how the assembler choice but also the assembly strategy or polishing strategy can affect metagenomic reconstruction results when using SR and LR data. The LR assembly approaches studied herein include polishing using SR and LR, whereas the HY assembly approaches construct an initial SR assembly graph, followed by graph traversal using LR and subsequent polishing using SR sequencing data. This distinction is important due to recent developments which leverage LR and SR reads together for the assembly. Furthermore, owing to the existence of established benchmarks [20] for SR/LR/HY assemblers, our analyses specifically address the influence of assemblies on functional discrepancies, which remain understudied thus far. Here, we reveal that sample diversity, along with assembly-mediated effects influence the prediction of genes and proteins. This causes discrepancies between the assemblies, thereby highlighting the potential for complementary means to validate these predictions. The observed discrepancies included conserved and also functionally relevant genes (rRNA and AMR genes, respectively), potentially impacting phylogenetic as well as functional studies. Besides software-driven differences, e.g. assembler choice, the extent of the differences in the metagenomic reconstruction approaches will also depend on associated costs and the thus achievable sequencing depth [21], complexity/composition of the sample and other factors, such as DNA extraction approaches [22], or library preparation methods [23], especially for low-abundance organisms. In addition to our newly generated human-borne multi-omic data (GDB), we used publicly available SR and LR metagenomic data originating from the same respective sample (Zymo, NWC or Rumen). The limited number of samples in this study is due to the limited availability of SR and LR sequence data and do not necessarily represent the extent of diversities across several metagenomic datasets. Although few studies exist to date that have published SR and LR data of the same sample, we expect more datasets to become available in the future due to the advantages that LR data brings for metagenomic reconstructions. To evaluate discrepancies in assembly approaches, we propose a reference-independent approach to identify high-confidence genomic reconstructions by combining metagenomic, metatranscriptomic and metaproteomic data. The appropriate coverage of metagenomic assemblies via metatranscriptomic reads and the potential presence of peptides mapping to the respective gene and proteins of interest indicate a validated transcription and translation, respectively. Overall, we show that the sequencing approach and assembly strategy can have a significant impact on the characterization of the microbiome’s functional potential and demonstrate the added value of multi-omic strategies for reconstruction quality evaluation, i.e. going beyond their original purpose, to resolve the functional microbiome.

## Materials and methods

### Sample origin and collection

The datasets (Zymo, NWC, Rumen) used herein were acquired from previously published reports regarding the utility of LR sequencing (Supplementary Table S2), with concomitant SR and LR sequencing data. The human fecal samples were freshly collected from a healthy volunteer (GDB) and immediately flash-frozen in liquid nitrogen. The samples were stored at −80°C until they were processed for biomolecular extraction of DNA, RNA and proteins.

### Biomolecular extraction

To obtain high-molecular weight (HMW) DNA, we followed the protocol proposed recently [8], with minor modifications. Frozen stool sample was weighed out in triplicates, to 0.7 g and aliquoted into phase-lock gel tubes (Fisher Scientific, Waltham, MA), along with a 4 mm stainless steel grinding balls (RETSCH 22.455.0003). The sample was subsequently suspended in 500 μl phosphate buffered saline (PBS) (Fisher Scientific, Waltham, MA) with brief gentle vortexing at 10 s intervals repeated five times. Thereafter, 5 μl of lytic enzyme solution (Qiagen, Hilden, Germany) was added and the samples were mixed by gentle inversion six times, and then incubated for 1 h at 37°C; 12 μl 20% (w/v) sodium dodecyl sulfate (SDS) (Fisher Scientific, Waltham, MA) was added followed by 500 μl phenol:chloroform:isoamyl alcohol at pH 8 (Fisher Scientific, Waltham, MA). The samples were gently vortexed for 5 s, and then centrifuged at 10 000 *g* for 5 min. The aqueous phase was decanted into a new 2 ml tube. Next, the DNA was precipitated with 90 μl 3 M sodium acetate (Fisher Scientific) and 500 μl isopropanol (Fisher Scientific). After slowly inverting three times, samples were incubated at room temperature for 10 min, followed by centrifugation for 10 min at 10 000 *g*. The supernatant was removed, and the pellet was washed twice with freshly prepared 80% (v/v) ethanol (Fisher Scientific). Washing was done by adding 1 ml of 80% EtOH, followed by centrifugation for 10 min at 10 000 *g*. The pellet was then air dried with heating for 10 min at 37°C or until the pellet was matte in appearance, and then resuspended in 100 μl nuclease-free water (Ambion, ThermoFisher Scientific, Waltham, MA). To the pellet, 1 ml Qiagen buffer G2, 4 μl Qiagen RNase A at 100 mg/ml and 25 μl Qiagen Proteinase K were added. The samples were then gently inverted three times and incubated for 90 min at 56°C. After the first 30 min, pellets were dislodged by a single gentle inversion. During the 90 min incubation, one Qiagen Genomic-tip 20/G column per triplicate sample was equilibrated with 1 ml Qiagen buffer QBT and allowed to empty by gravity flow. Samples were gently inverted twice, applied to columns and allowed to flow through. Three stool extractions (triplicates for each sample) were combined per column. Columns were then washed with 3 ml Qiagen buffer QC, where 1 ml of QC buffer was added each time and allowed to drain the column. Next, the column was placed in a new sterile 1.5 ml Eppendorf tube and the DNA was then eluted with 1 ml of Qiagen buffer QF prewarmed to 56°C. The eluted DNA was then precipitated by addition of 700 μl isopropanol and incubated at room temperature for 10 min, followed by inversion and centrifugation for 15 min at 10 000 *g*. The supernatant was carefully removed by pipette, and pellets were washed with 1 ml 80% (v/v) ethanol (washing = add 1 ml EtOH, centrifuge for 10 min at 10 000 *g*). Residual ethanol was removed by air drying 10 min at 37°C, followed by resuspension of the pellet in 100 μl water overnight at 4°C without agitation of any kind. The pooled sample was quantified using the Qubit Broad-Range DNA concentration kit and was estimated at 323.35 ng/μl with an OD_260/280_ = 1.85. The extracted HMW DNA was used for both SR and LR sequencing. RNA was extracted from an aliquot of the same fecal sample using PowerMicrobiome RNA isolation kit (cat. no. 26000-50, MoBio) as suggested by the manufacturer. For the protein extractions, a modified protocol based on a previously established sequential extraction method [24] was used. Briefly, proteins were precipitated by adding one volume of All-Prep Protein (APP) Buffer to the flow-through from an independent RNA purification, followed by mixing and incubation for 10 min at room temperature. After incubation, the mixture was centrifuged for 10 min at 12 000 *g* and the pellet was washed twice in 70% ethanol, with 1 min centrifuge cycles at 12 000 *g*, and dried at room temperature for 7 min after removing excess ethanol. The pellet was then dissolved in 100 μl ALO buffer and incubated for 5 min at 95°C. After complete dissolution and denaturation of the protein, the sample was cooled to room temperature and centrifuged for 1 min at 12 000 *g*, from which the supernatant was collected for downstream protein analysis.

### Sequencing

#### SR sequencing

The DNA sample was subjected to random shotgun sequencing. The sequencing library was prepared using KAPA HyperPlus Kit (cat. no. 07962401001, Roche) for the GDB fecal sample using the protocol provided with the kit. Enzymatic fragmentation time was 15 min to aim for 350 bp average size. There was no additional polymerase chain reaction amplification of the prepared library.

RNA sample for metaT analysis was subjected to rRNA depletion using the QIAseq FastSelect 5S/16S/23S kit (cat. no. 335921, Qiagen) for the GDB fecal sample. Library preparation of rRNA-depleted RNA was done using TruSeq Stranded mRNA library preparation kit (cat. no. 20020594, Illumina) according to the protocol provided by the manufacturer with the exception of omitting the initial steps for mRNA pull down.

Both metaG and metaT libraries were quantified using Qubit HS assay (Invitrogen) and their quality was assessed on a Bioanalyzer HS chip (Agilent). We used the NextSeq500 (Illumina) instrument to perform the sequencing using 2 × 150 bp read length at the Luxembourg Centre for Systems Biomedicine (LCSB) Sequencing Platform.

#### LR sequencing

DNA library for the fecal sample was size selected using AMpure beads for longer fragments. The DNA was sheared using a G-tube (cat. no. 520079, Covaris) aiming for 8 kb average size according to the protocol provided by the manufacturer. Library preparation for LR sequencing was done using the genomic DNA ligation kit (SQK-LSK109) according to the protocol provided by the manufacturer using a MinION R9.4.1 flowcell. Once all the library loaded on the flowcell was finished, the library was reloaded after either flowcell wash or nuclease flush. In total, the library was loaded four times to achieve 16 Gbp of sequencing data for this fecal sample (Supplementary Table S3).

### Data analysis

Snakemake (v. 5.18.1) [25] was used to implement the analysis workflow. We provide a brief description of the most important steps in the following.

### Sequence data preprocessing

#### Short reads

The raw SRs were trimmed and preprocessed with fastp (v. 0.20.0) [26] with a minimum length of 40 bp. FastQC (v. 0.11.9) [27] reports were generated from the processed FASTQ files. MetaT SRs from the GDB sample were filtered by discarding reads mapping to rRNA gene references included in the repository of SortMeRNA [28] (v4.2.0-10-g1358b9b, https://github.com/biocore/sortmerna) using BBDuk from the BBMap toolkit (v.38.86, kmer length set to 31 bp) [29]. In addition, for the GDB sample, reads mapping to the human genome (GCF_000001405.38_GRCh38.p12) were removed using BBDuk (kmer length set to 31 bp, input and output quality encoding offset set to 33).

#### Long reads

For each sample except NWC, single-FAST5 files were converted to multi-FAST5 files using single_to_multi_fast5 from ont-fast5-api (v. 3.1.5), the resulting files were basecalled using guppy on a GPU node (v. 3.6.0 + 98ff765, configuration file dna_r9.4.1_450bps_modbases_dam-dcm-cpg_hac.cfg, disabled transmission of telemetry pings, chunk size of 1000, 8000 records per FASTQ file) and concatenated into a single FASTQ file. For NWC, no FAST5 were available and, thus, only the provided FASTQ file was used for the analysis. Nanostat (v. 1.1.2) [30] reports were created from the FASTQ files using default parameters. As for the SRs, LRs of the GDB sample were filtered to remove reads mapping to the human genome (GCF_000001405.38_GRCh38.p12) using the same parameters.

### Metagenomic assembly

#### Short reads

SR assemblies were done using preprocessed reads and MEGAHIT or metaSPAdes. MEGAHIT (v. 1.2.9) [31] was run using default parameters; metaSPAdes (v. 3.14.1) [32] was run using kmer lengths 21, 33, 55 and 77 bp.

#### Long reads

LR assemblies were done using Flye and Raven. Flye (v. 2.8.1) [33] was run by providing the (processed) LRs in a FASTQ file (input parameter ‘--nano-raw’) and with the flag ‘--meta’. Raven (v. 1.2.2) [34] was run with default parameters. Assemblies were polished using LR and SR: one round of Racon (v. 1.4.13) [35] with LRs using the flag ‘-include-unpolished’ where reads were mapped to contigs using BWA MEM (v. 0.7.17) [36] with the option ‘-x ont2d’ and processed using samtools (v. 1.9); four rounds of Racon with SRs using the flag ‘--include-unpolished’ where reads were mapped to contigs using Burrows-Wheeler Aligner (BWA-MEM) and processed using samtools; one round of Medaka (v. 0.8.1) [37] with LRs using the model ‘r941_min_high’.

#### Hybrid

HY assemblies, i.e. using SR and LR together, were done using metaSPAdes and OPERA-MS. SPAdes was run with the flag ‘--meta’ and the same k-mer lengths as the SR assemblies by additionally providing the LRs using the input parameter flag ‘--nanopore’. OPERA-MS (v. v0.8.2-63-gc18b4f3) [15] was run using paired SRs, LRs and the SR assemblies created by MEGAHIT and metaSPAdes, respectively, using minimap2 [38] as the LR mapper. The assemblies were polished by running five rounds of Racon with SRs as described for the LR assemblies. If not stated otherwise, only polished contigs were used for the LR and HY assemblies in the following analysis steps.

### Mapping rate and assembly coverage

For the mapping rate, the used reads were mapped back to the contigs and processed using BWA MEM and samtools in the same way as described above when polishing the LR and HY assemblies using Racon. For HY assemblies, both LR and SR were mapped to the polished contigs and the BAM files were merged using samtools. For the sample GDB, metaT SRs were also separately mapped to the (polished) contigs. Mapping statistics were computed from the BAM files using samtools’ options ‘flagstat’, to determine the number of reads mapping back to the assemblies, and ‘idxstats’ for per-contig mapping information. For GDB, metaT per-base coverage was computed for each assembly from the BAM files using bedtools (v. 2.29.2) [39] (utility ‘genomecov’ with the parameter ‘-d’).

### Assembly annotation

For each sample and assembly, protein prediction was done using Prodigal (v. 2.6.3) [40] using the option ‘-p meta’; the keyword ‘partial’ in the headers of the obtained protein FASTA files was used to distinguish complete and partial proteins. Known antibiotic resistance factors were searched in the predicted proteins (after discarding the stop codon symbol ‘*’ from the FASTA files) by running RGI (v. 5.1.1) [41] together with the CARD database (v. 3.1.0) [42] and DIAMOND (v. 0.8.36) [43] for protein alignments. Loose hits flagged as ‘nudged’ by the tool were highlighted as such (i.e. as ‘nudged’) in the downstream analysis.

The tool barrnap (v. 0.9) [44] was run to predict rRNA genes on assembly contigs using the four provided databases of bacterial, archaeal, metazoan mitochondrial, and eukaryotic rRNA genes, respectively. Predictions containing the word ‘partial’ in their product annotation in the obtained General Feature Format (GFF) files were considered as partial hits.

### Analysis

Assembly statistics were computed by running metaQUAST (v. 5.0.2) [45] without using any genome references, setting the minimum contig length to 0 bp and retrieving the statistics for the contig length thresholds of 0, 1000, 2000 and 5000 bp subsequently. Per sample, assemblies were compared using Mash (v. 2.2.2) [46]: sketches were computed per assembly using a k-mer length of 31 bp and a sketch size of 100 000, and pairwise distances were then estimated. Per sample, proteins from all assemblies were clustered using MMseqs2 (v. 12.113e3) [47]. First, a database was created from a concatenated FASTA file of protein sequences (‘--dbtype 1’). Then, option ‘linclust’ with default parameters was used to perform the clustering and the obtained files were converted to tables using option ‘createtsv’. DIAMOND (v. 0.9.25) [43] with the option ‘blastp’ and default parameters was used to align the predicted proteins against the UniProtKB/TrEMBL database (downloaded and created on 24 August 2019 from http://ftp.uniprot.org/pub/databases/uniprot/current_release/knowledgebase/complete/, archive uniprot_trembl.fasta.gz) [48]. The created DIAMOND alignment archive (DAA) files were converted to tables using option ‘view’ and the parameter ‘--max-target-seqs 1’. When processing the hits, these were sorted per query and e-value in ascending order and only the first hit was used. For GDB and metaT, using the per-base coverage information computed for each assembly, the average coverage was computed for the corresponding gene sequences of each predicted protein.

### MS/MS acquisition and metaproteomic analysis

One microgram of extracted proteins was denatured and loaded on a SDS gel to produce one gel band. The reduction, alkylation and tryptic digestion of the proteins into peptides were performed in-gel. The tryptic peptides were extracted from the gel and desalted prior to mass spectrometry analysis. Peptides were analyzed using a nano Liquid Chromatography-Mass Spectrometry/Mass Spectrometry (nanoLC-MS/MS) system (120 min gradient) connected to a Q-Exactive HF orbitrap mass spectrometer (Thermo Scientific, Germany) equipped with a nano-electrospray ion source. The Q-Exactive mass spectrometer was operated in data-dependent mode and the 10 most intense peptide precursor ions were selected for fragmentation and MS/MS acquisition.

For each assembly separately and for all assemblies together, the FASTA file of predicted proteins was concatenated with a common Repository of Adventitious Proteins (cRAP) database of contaminants [49] and with the human UniProtKB Reference Proteome prior metaproteomic search. In addition, reversed sequences of all protein entries were concatenated to the databases for the estimation of false discovery rates (FDRs). The search was performed using SearchGUI-3.3.20 [50] with the X!Tandem [51], MS-GF+ [52] and Comet [53] search engines and the following parameters: trypsin was used as the digestion enzyme and a maximum of two missed cleavages was allowed. The tolerance levels for matching to the database were 10 ppm for MS1 and 0.02 Da for MS2. Carbamidomethylation of cysteine residues was set as a fixed modification and protein N-terminal acetylation and oxidation of methionines was allowed as variable modification. Peptides with length between 7 and 60 amino acids and with a charge state composed between +2 and +4 were considered for identification. The results from SearchGUI were merged using PeptideShaker-1.16.45 [54] and all identifications were filtered in order to achieve a protein FDR of 1%.

### Plots

Figures were generated in R (v. 4.0.2, https://www.r-project.org/) using, *inter alia*, Pheatmap (v. 1.0.12, https://github.com/raivokolde/pheatmap) for heatmap plots, UpSetR (v. 1.4.0) [55] for intersection plots, ggplot2 (v 3.3.2) [56] and its various extensions for other plot types, color palettes from the viridis (v. 0.5.1, https://github.com/sjmgarnier/viridis) and ggsci (v. 2.9, https://github.com/road2stat/ggsci) packages and the patchwork package (v. 1.1.1, https://github.com/thomasp85/patchwork) for combining plots.

**Key Points**

- Sequencing and assembly approach affect gene and protein inference.
- Meta-omics enable critical assessment of metagenome reconstructions.
- Reference-independent solution which exploits synergies of next-generation and third-generation sequencing approaches that results in improved integration of meta-omics data.

## Authors’ contributions

S.B.B., V.G. and C.C.L. designed the study. S.B.B. and R.H. performed the biomolecular extractions, whereas R.H. performed the metagenomic and metatranscriptomic sequencing. V.G., S.B.B., L.deN. and C.C.L. analyzed the data. B.J.K. performed the metaproteomic analyses. P.M., M.C. and P.W. provided critical feedback and insights. All authors contributed to the writing and revision of the manuscript.

## Supplementary Material

Supplementary_Table_2_Datasets_bbab330

Supplementary_Table_3_ONT_Run_Statistics_bbab330

fig_quast_bbab330

fig_mappability_bbab330

fig_mappability_gdb_metat_bbab330

fig_mash_bbab330

fig_diamond_db_bbab330

fig_barrnap_bbab330

tab_rgi_gdb_bbab330

## Data Availability

Processed sequencing data of the GDB sample is available under BioProject accession PRJNA723028 (Biosamples: metag_sr: SAMN18797629, metat_sr: SAMN18797630 and metag_lr: SAMN18797631). Metaproteomics data of the GDB sample is available at ProteomeXchange under accession PXD025505. The code used for the analysis is available at https://doi.org/10.17881/sgzt-ad12 (v1.0) and supplementary data of relevant results is available at https://doi.org/10.6084/m9.figshare.14447559.

## Abbreviations

SR, short reads; LR, long reads; HY, hybrid (approach/assembly); metaG, metagenomic (data); metaT, metatranscriptomic (data); metaP, metaproteomic (data); AMR, antimicrobial resistance; rRNA, ribosomal RNA; ARO, Antibiotic Resistance Ontology; CDS, coding sequence; HMW, high-molecular weight; FDR, false discovery rate; indels, insertions/deletions; nr, nonredundant

## Notes

**Valentina Galata** is a Research Associate in the Systems Ecology Group, Luxembourg Centre for Systems Biomedicine, University of Luxembourg, Esch-sur-Alzette, Luxembourg.

**Susheel B. Bhusi** is a Research Associate in the Systems Ecology Group, Luxembourg Centre for Systems Biomedicine, University of Luxembourg, Esch-sur-Alzette, Luxembourg.

**Benoît J. Kunath** is a Research Associate in the Systems Ecology Group, Luxembourg Centre for Systems Biomedicine, University of Luxembourg, Esch-sur-Alzette, Luxembourg.

**Laura de Nies** is a PhD student in the Systems Ecology Group, Luxembourg Centre for Systems Biomedicine, University of Luxembourg, Esch-sur-Alzette, Luxembourg.

**Magdalena Calusinska** is a Senior Research & Technology Associate in the BioSystems and Bioprocessing Engineering group, Luxembourg Institute of Science and Technology, Belvaux, Luxembourg.

**Rashi Halder** is a Research Associate in the Systems Ecology Group and in the LCSB Sequencing Platform, University of Luxembourg, Esch-sur-Alzette, Luxembourg.

**Patrick May** is a Senior Researcher and the Head of the Genome Analysis group, Bioinformatics Core, Luxembourg Centre for Systems Biomedicine, University of Luxembourg, Esch-sur-Alzette, Luxembourg.

**Paul Wilmes** is the Head of the Systems Ecology Group, Luxembourg Centre for Systems Biomedicine, University of Luxembourg, Esch-sur-Alzette, Luxembourg, and a Professor in the Department of Life Sciences and Medicine, Faculty of Science, Technology and Medicine, University of Luxembourg, Esch-sur-Alzette, Luxembourg.

**Cedric C. Laczny** is a Research Scientist in the Systems Ecology Group, Luxembourg Centre for Systems Biomedicine, University of Luxembourg, Esch-sur-Alzette, Luxembourg.
