# Supplementary figures and images for: Functional meta-omics provide critical insights into long- and short-read assemblies
Source: Brief Bioinform. 2021 Aug 27;22(6):bbab330. doi: 10.1093/bib/bbab330 (PMC8575027; doi:10.1093/bib/bbab330)

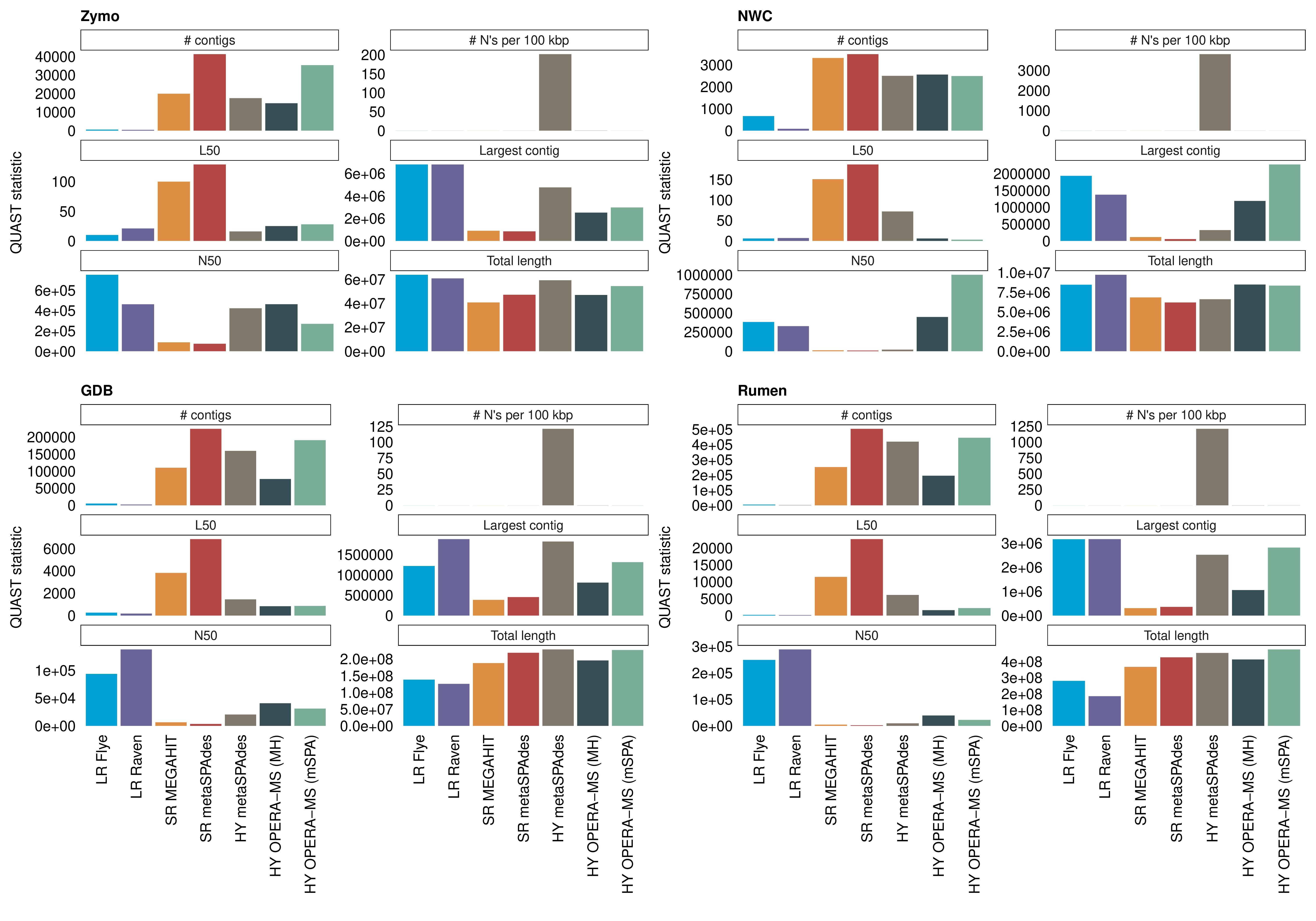

Supplement: fig_quast_bbab330 [file fig_quast_bbab330.jpeg]

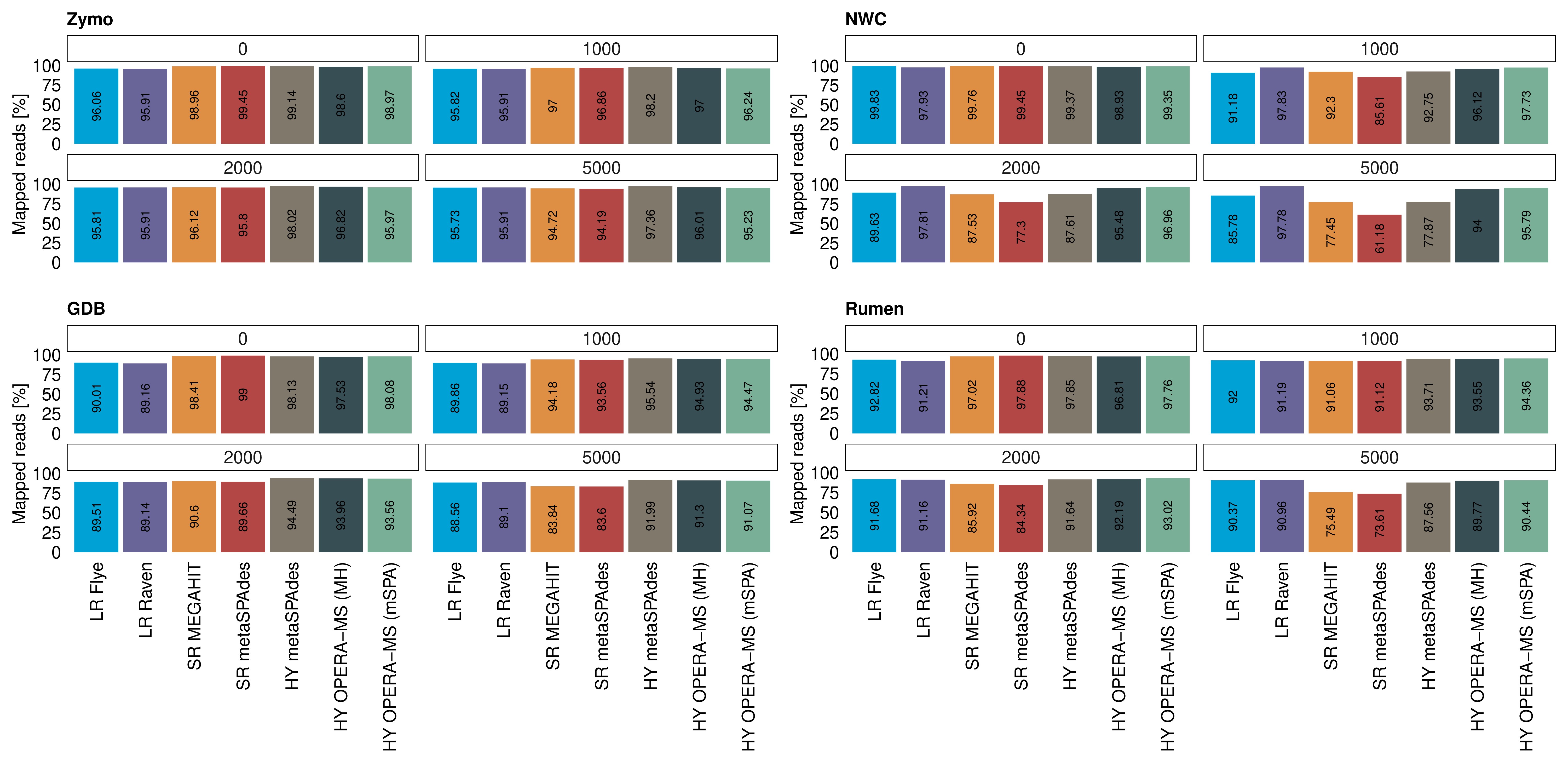

Supplement: fig_mappability_bbab330 [file fig_mappability_bbab330.jpeg]

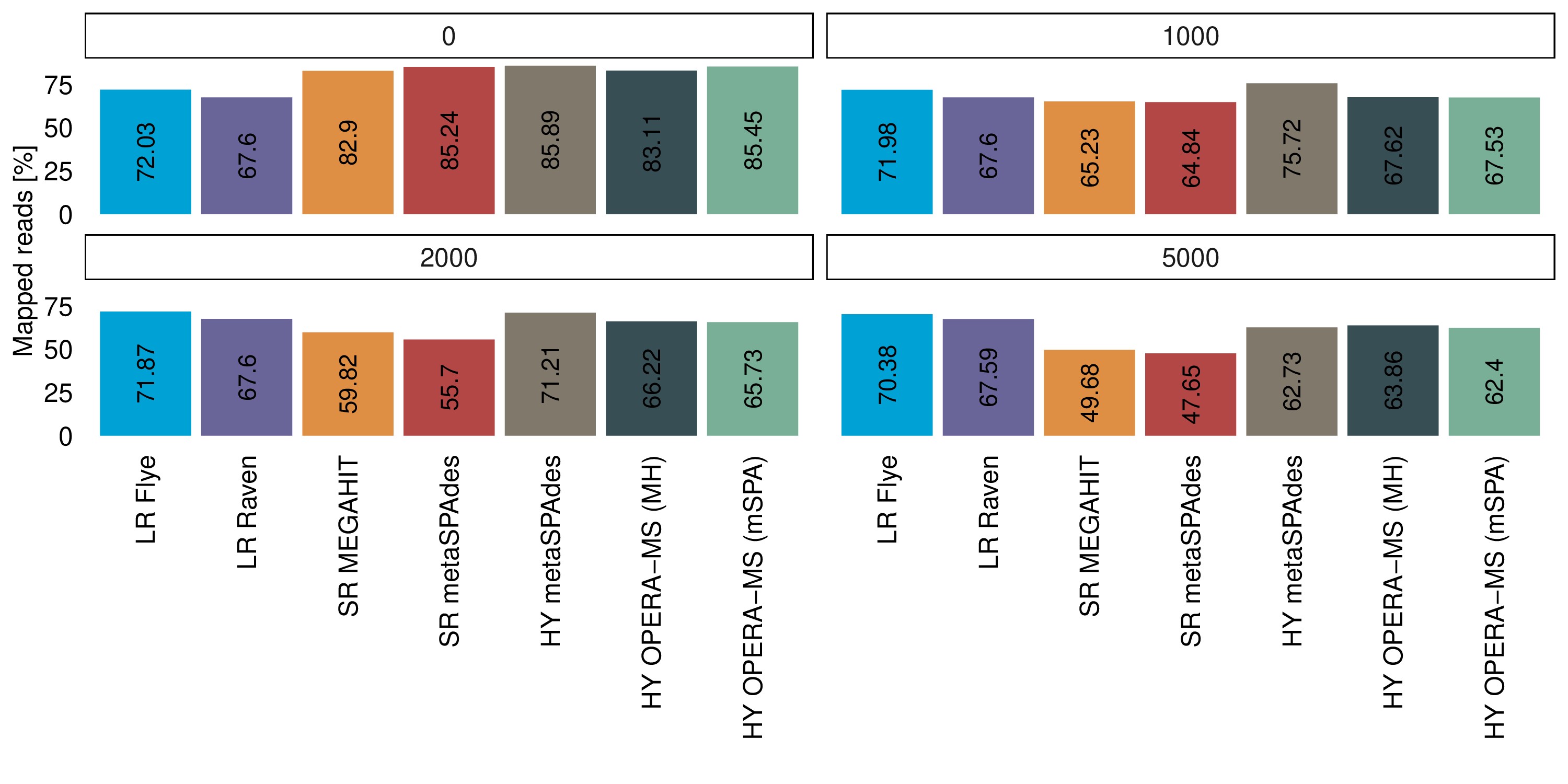

Supplement: fig_mappability_gdb_metat_bbab330 [file fig_mappability_gdb_metat_bbab330.jpeg]

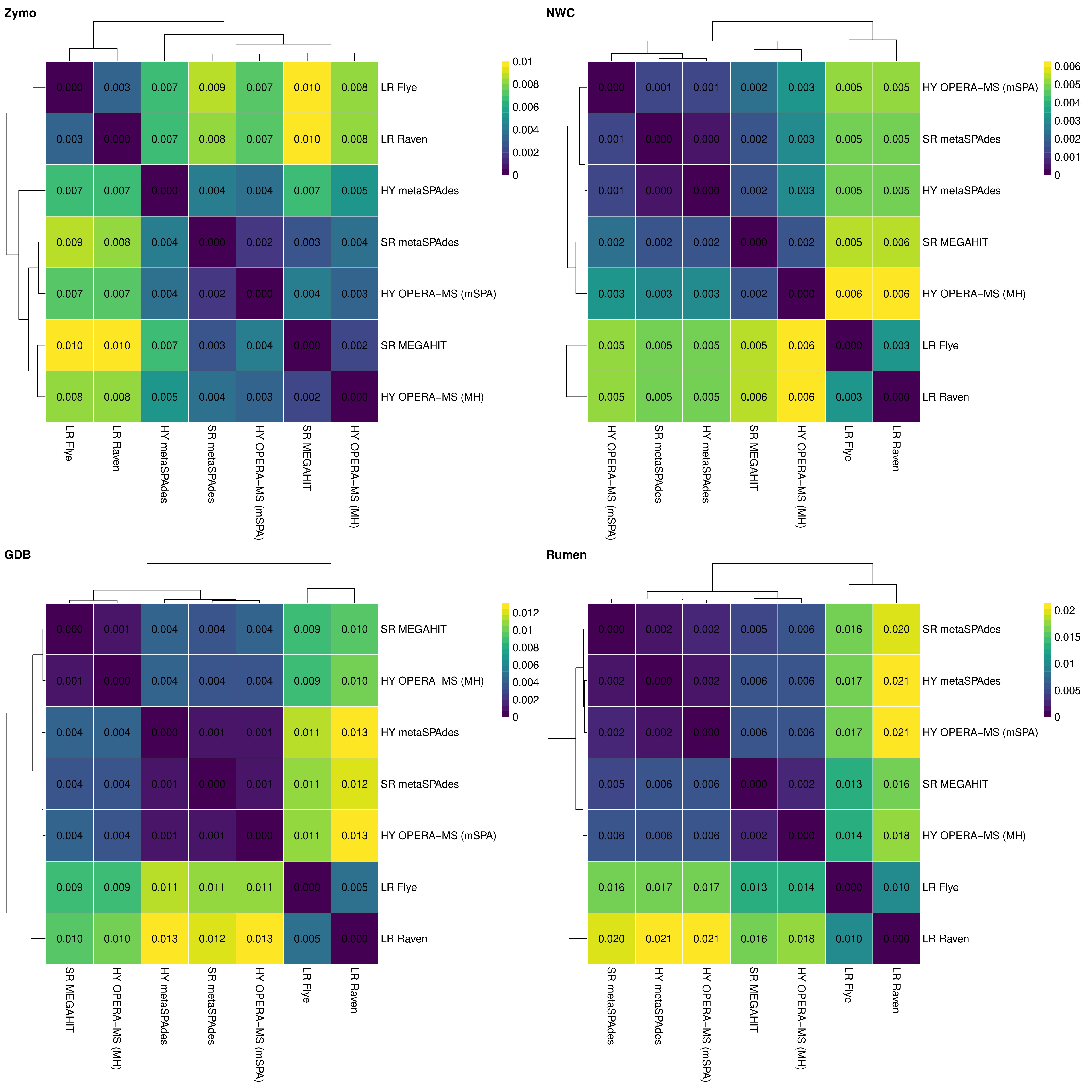

Supplement: fig_mash_bbab330 [file fig_mash_bbab330.jpeg]

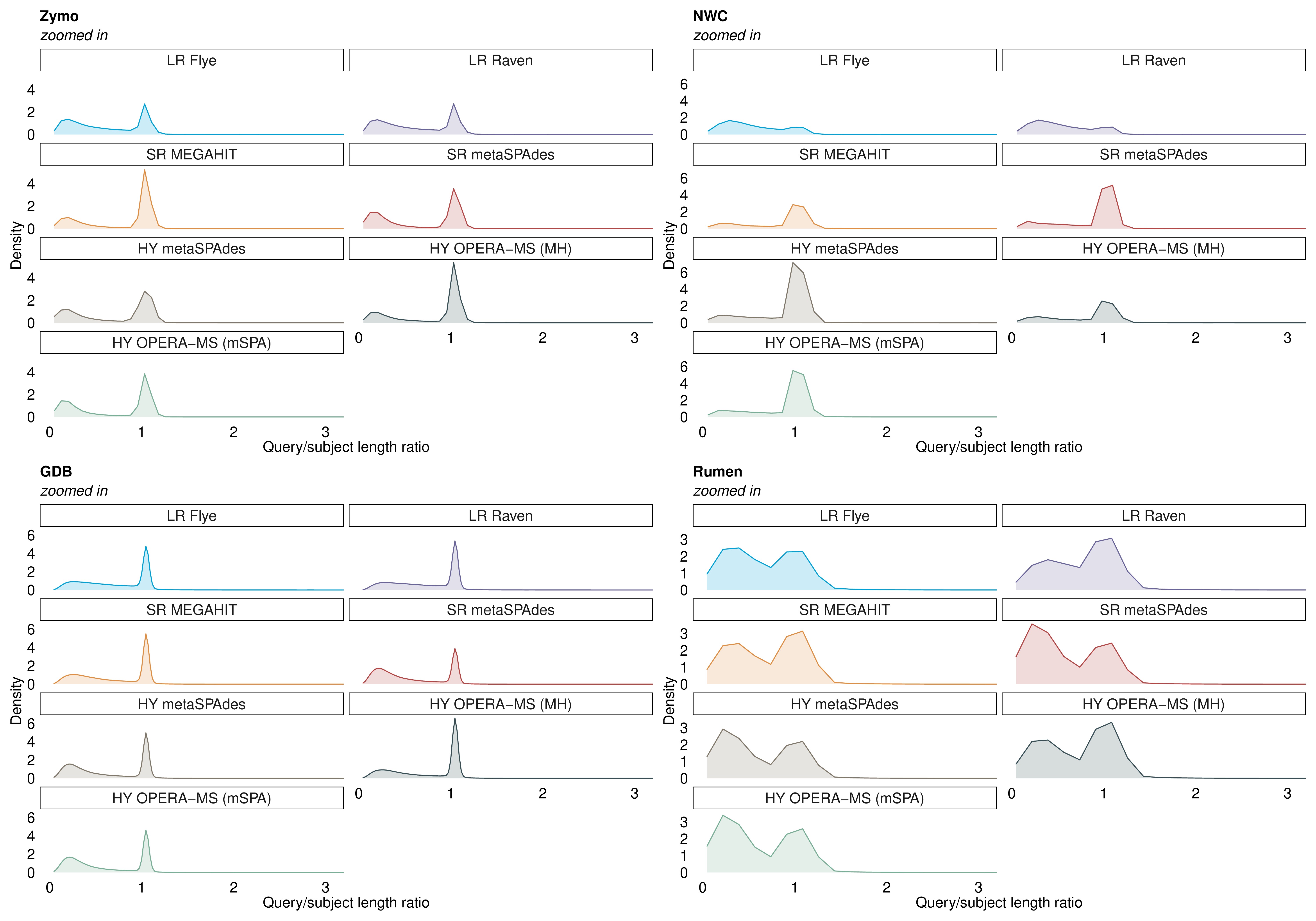

Supplement: fig_diamond_db_bbab330 [file fig_diamond_db_bbab330.jpeg]

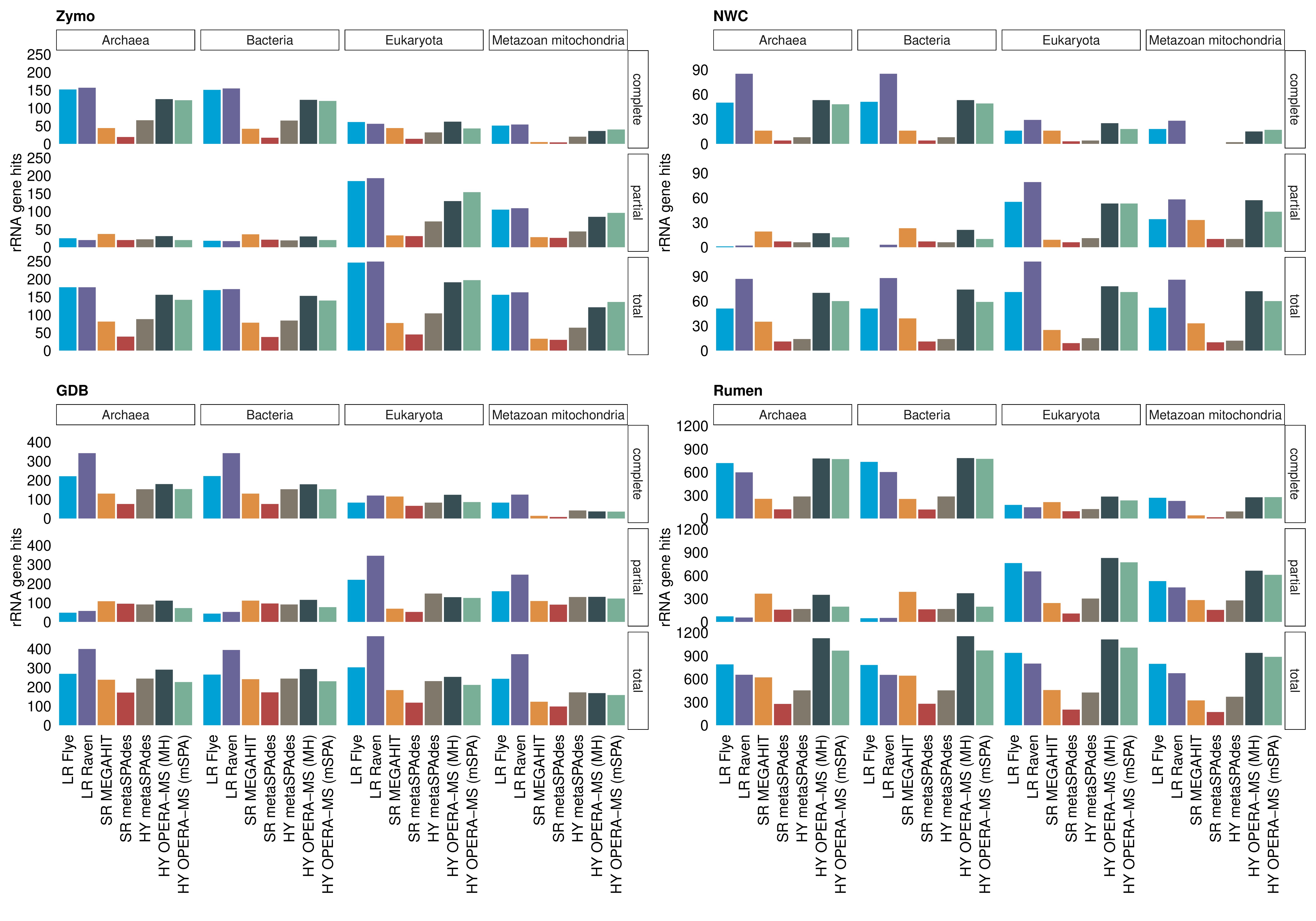

Supplement: fig_barrnap_bbab330 [file fig_barrnap_bbab330.jpeg]
